# Mini-Mental State Examination: Optimal Cut-Off Levels for Mild and Severe Cognitive Impairment

**DOI:** 10.3390/geriatrics8010012

**Published:** 2023-01-12

**Authors:** Francesco Salis, Diego Costaggiu, Antonella Mandas

**Affiliations:** Department of Medical Sciences, and Public Health, University of Cagliari, SS 554 Bvio Sestu, 09042 Monserrato, Italy

**Keywords:** dementia, memory, mild cognitive impairment (MCI), Mini-Mental State Examination (MMSE), Repeatable Battery for the Assessment of Neuropsychological Status (RBANS), screening

## Abstract

Considering the need to intercept neurocognitive damage as soon as possible, it would be useful to extend cognitive test screening throughout the population. Here, we propose differential cut-off levels that can be used to identify mild and severe cognitive impairment with a simple and widely used first-level neurocognitive screening test: the Mini-Mental State Examination (MMSE). We studied a population of 262 patients referred for cognitive impairment testing using the MMSE and Repeatable Battery for the Assessment of Neuropsychological Status (RBANS), a neuropsychological battery. The sample consisted of 262 participants with mean age 73.8 years (60–87), of whom 154 (58.8%) women. No significant gender-related differences in cognitive ability were identified. The two tests (MMSE and RBANS) showed a moderate correlation in identifying cognitive deficit. We used RBANS as a categorial variable to identify different degrees of cognitive impairment. Youden’s J indexes were used to consider the better sensitivity/specificity balance in the 24-point cut-off score for severe cognitive deficit, 29.7-point score for mild cognitive deficit, and 26.1-point score for both mild and severe cognitive deficit. The study shows that the MMSE does not identify early cognitive impairment. Though different cut-offs are needed to discriminate different impairment degrees, the 26.1-point score seems to be preferable to the others.

## 1. Introduction

The 2019 *World Alzheimer Report* [1] underlines the fact that the current population affected by neurocognitive disorders (including mild cognitive impairment, which represents a 3–4 relative risk of dementia), which amounts to around 50 million people, is destined by 2050 to reach more than triple that figure (152 million) due to the increase in the average global median age. The ageing of the population inevitably leads to an increase in geriatric syndromes, such as anemia [2], neurocognitive disorders [1], frailty, and polypharmacotherapy [3].

A report published in 2019 [4] showed cautious optimism about the possibility of having, in the near future, a therapy able to slow the progression of Alzheimer’s disease but also lamented the lack of tools capable of detecting impairment in time for the therapy to work.

It therefore seems appropriate to focus on first-level neurocognitive tests in order to enable patients to seek help from health professionals before their neurocognitive status is impaired. Incidentally, it should be noted that other tools for the early identification of damage to central nervous system are available [5].

Several studies are to be found in the scientific literature, which refer to Mini-Mental State Examination (MMSE) and also Montreal Cognitive Assessment (MoCA), comparing their capacity to detect cognitive impairment. A 2016 meta-analysis [6] concluded that MoCA seems to be more sensitive and specific than MMSE for the detection of mild deterioration in people aged over 60. However, unlike MMSE, neither in clinical practice nor in the literature is MoCA widely present or employed. A 2019 meta-analysis [7] makes it clear that, despite the fact that MoCA and other tests seem to be more sensitive, MMSE is the most frequently used worldwide. One of the most widely discussed topics on MMSE is the interpretation of the cut-off score used to identify cognitive impairment. In the literature, it is debated as to which guarantees the best sensitivity/specificity ratio [8]. Nonetheless, the test can be used in patient follow-up to assess the effectiveness of any treatment [9,10,11].

Nevertheless, neuropsychological batteries aid the diagnosis of neurocognitive disorders. The Repeatable Battery for the Assessment of Neuropsychological Status (RBANS) is one of them. It offers the possibility of identifying different degrees of neurocognitive deficit and is also useful for characterizing neurocognitive disorders of different etiologies. For example, this battery is not only proven to have a sufficiently high accuracy diagnosis-wise [12], but it is also useful for detecting HIV-associated neurocognitive disorders [13,14]. The RBANS’ areas of application also extend to other causes of cognitive impairment, such as alcohol dependence [15], post-traumatic stress disorder [16], and progressive supranuclear palsy [17].

Given the need to intercept neurocognitive damage as soon as possible, the primary aim of the study was to understand MMSE’s accuracy in identifying cognitive impairment.

The secondary aim of the study was to identify MMSE’s cut-off levels offering the optimal sensitivity and specificity in identifying both mild and severe cognitive impairment.

## 2. Methods

- Design of the study
  We performed a cross-sectional study including subjects who were consecutively evaluated at the Geriatric Outpatient Service of Cagliari University Hospital between January and August 2019. The accuracy of MMSE was assessed in comparison with RBANS.
  Inclusion criteria: age ≥ 60 years and presenting with subjective cognitive impairment complaints.
  Exclusion criteria: age < 60 years; not previously subjected to MMSE and/or RBANS; not presenting with subjective cognitive impairment complaints; and consent not provided.
  A sum of 262 subjects met the inclusion criteria.
  The sample were subjected by trained geriatricians to the following:
  - RBANS
  First used as a diagnostic tool, RBANS [18,19] subsequently became a screening tool, which, unlike others, can draw a profile of the various cognitive domains. RBANS evaluates five cognitive domains (immediate memory, visuospatial/constructional abilities, language, attention, and delayed memory), each with an index score (RBANS-IS). The range of the five index scores’ sum is from 200 to 800. This sum is converted into the total scale index (RBANS-TIS). To avoid the influences of age differences, conversion tables are available. A RBANS-TIS (and also RBANS-IS) between 85 and 70 (1 Standard Deviation (SD) below the mean) indicates a probable deficit, while a score of <70 (2 SD below the mean) likely indicates cognitive impairment.
  - MMSE

MMSE [20,21] is a quick and simple screening tool used to evaluate five cognitive domains (orientation, immediate memory, attention, delayed memory, and language). The sum of the scores obtained for each of them gives a total score ranging from 0 to 30. To avoid the influences of age and schooling differences, conversion tables are available.

- Statistical analysis

Quantitative variables were expressed as means ± SD. The scores’ correlation was studied using Pearson’s correlation coefficient (r). MMSE’s internal consistency was studied using Cronbach’s alpha. MMSE’s performance was studied using the area under the receiver operating characteristic (ROC) curve (AUC). Youden’s J statistic was used to identify the optimal cut-off levels according to the sensitivity, specificity, and positive and negative likelihood ratios (+LR, −LR).

The results are reported, indicating *p*-values in reference to 95% confidence intervals.

MedCalc software (Version 19.5, Ostend, Belgium) was used for the statistical analysis.

## 3. Results

The study included 262 participants with subjective cognitive impairment complaints (age: 73.8 ± 5.8 years (range: 60–87); gender: 154 (58.8%) women, 108 (41.2%) men). The characteristics of the enrolled subjects are shown in Table 1. There were no significant gender-related differences in the MMSE (*p* = 0.26) and RBANS (*p* = 0.78) scores.

According to RBANS, 66 people (25.2%) had adequate cognitive abilities (RBANS ≥ 85). Of the other 196 subjects, 79 (30.15% of the sample) had severe deficit (RBANS < 70), while a large group (187 subjects, 71.4%) scored MMSE ≥24.

**Figure 1:**
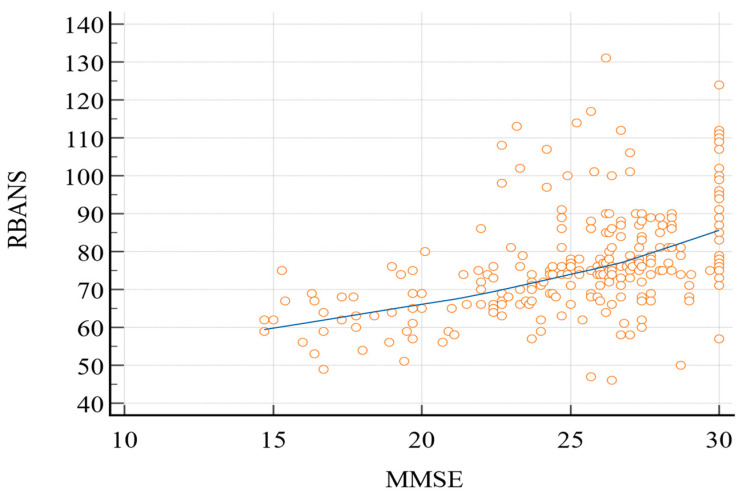
Pearson’s correlation coefficient (RBANS vs. MMSE). MMSE, Mini-Mental State Examination; RBANS, Repeatable Battery for the Assessment of Neurocognitive Status.

Pearson’s correlation coefficient (Figure 1) between the two tests was 0.49 (95% confidence interval (C.I.): 0.39–0.57; *p* < 0.0001).

We studied MMSE’s internal consistency using Cronbach’s alpha. The coefficient was 0.63 (95% lower confidence limit: 0.57), and the effect of the dropping variables ranged between 0.49 and 0.68 (Table 2).

RBANS was used as the “classification variable” to identify the presence (“1”) or absence (“0”) of cognitive impairment. The 85-point cut-off score was used to define mild deficit, and the 70-point cut-off score was used to define severe deficit.

**Figure 2:**
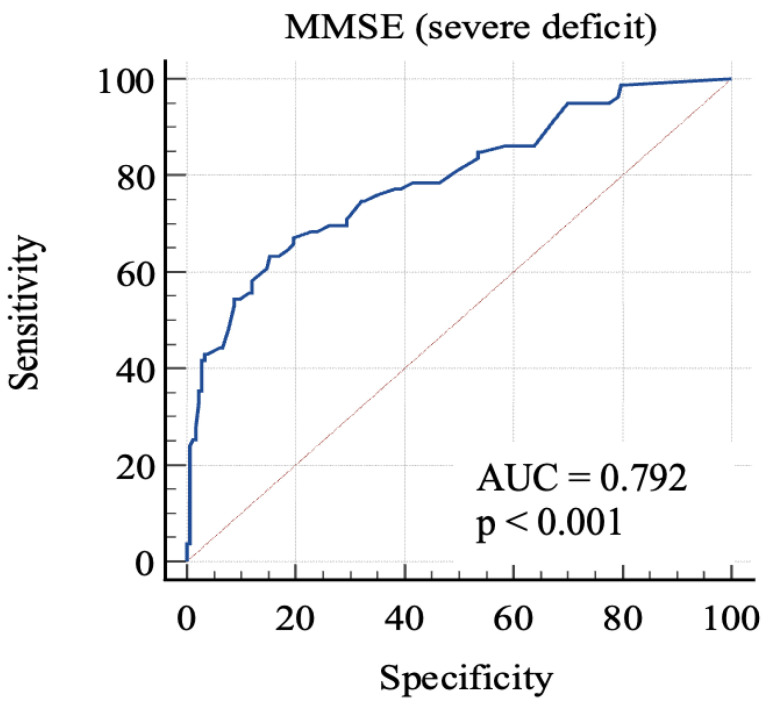
AUC (severe impairment). MMSE, Mini-Mental State Examination; AUC, area under the ROC curve.

For the first, we considered 0: RBANS ≥ 70 and 1: RBANS < 70. In this analysis (Figure 2), MMSE showed an AUC: 0.79 (standard error: 0.03, 95% C.I.: 0.74–0.84; *p* < 0.0001). Each cut-off score’s sensitivity, specificity, +LR, and −LR are shown in Table 3. Youden’s J index was 0.48 for the criterion of MMSE ≤ 24, with 63.3% sensitivity, 84.7% specificity, 4.14 +LR, and 0.43 −LR.

**Figure 3:**
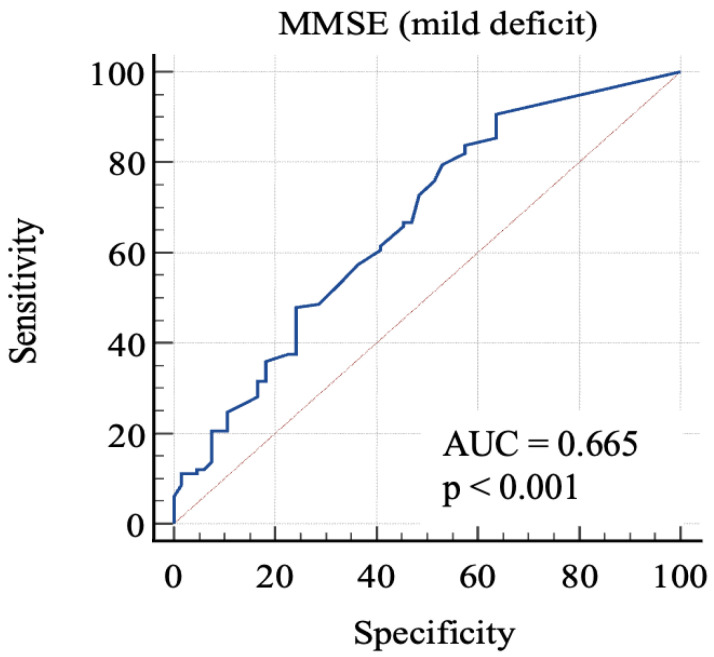
AUC (mild impairment). MMSE, Mini-Mental State Examination; AUC, area under the ROC curve.

Then, we considered 0: RBANS ≥ 85 and 1: RBANS < 85 and ≥70. In this analysis (Figure 3), MMSE showed an AUC: 0.665 (standard error: 0.04; 95% C.I.: 0.59–0.73; *p* = 0.0001). Each cut-off score’s sensitivity, specificity, +LR, and −LR are shown in Table 4. Youden’s J index was 0.27 for the criterion of MMSE ≤ 29.7, with 90.6% sensitivity, 36.4% specificity, 1.42 + LR, and 0.26 − LR.

**Figure 4:**
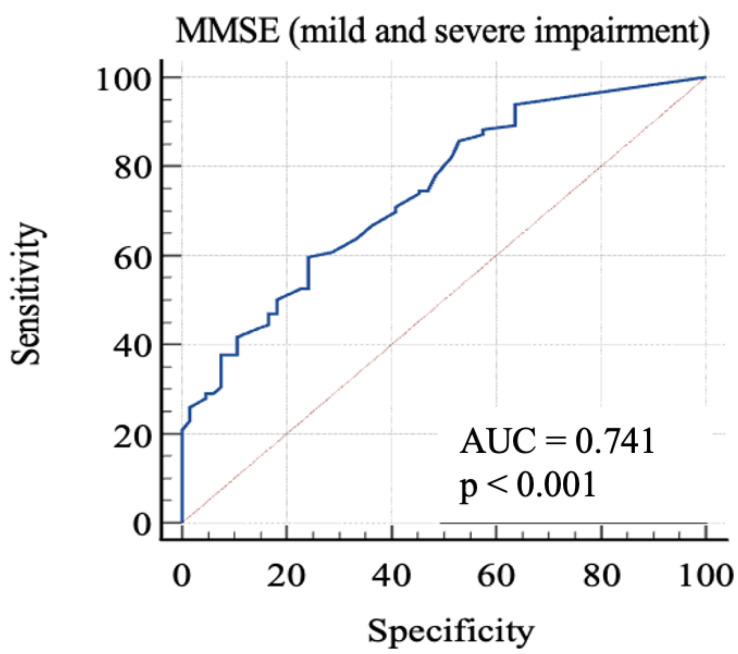
AUC (mild and severe impairment). MMSE, Mini-Mental State Examination; AUC, area under the ROC curve.

Finally, we considered 0: RBANS ≥ 85 and 1: RBANS < 85. In this analysis (Figure 4), MMSE showed an AUC: 0.74 (standard error: 0.03; 95% C.I.: 0.68–0.79; *p* < 0.0001). Each cut-off score’s sensitivity, specificity, +LR, and −LR are shown in Table 5. Youden’s J index was 0.35 for the criterion of MMSE ≤ 26.1, with 59.69% sensitivity, 75.76% specificity, 2.46 + LR, and 0.53 − LR.

The threshold was also studied using the different subsets of the sample after dividing it into quartiles according to the variable of “age” (Table 6). In the 1st quartile (≤70 years), Youden’s J index was 0.36 for the criterion of MMSE ≤ 29.7, with 88.89% sensitivity and 46.88% specificity (*p* = 0.0005). In the 2nd quartile (71–74), Youden’s J index was 0.36 for the criterion of MMSE ≤ 23.7, with 41.46% sensitivity and 94.44% specificity (*p* = 0.0058). In the 3rd quartile (75–78), Youden’s J index was 0.67 for the criterion of MMSE ≤ 26, with 67.24% sensitivity and 100% specificity (*p* < 0.0001). Finally, in the 4th quartile (≥79), the AUC showed non-significant results (*p* = 0.0809).

## 4. Discussion

In this study, we collected and examined data from a sample of 262 subjects referred for neurocognitive function assessment. The aims of the study were to analyze MMSE’s accuracy in identifying cognitive impairment by comparing it with a more sensitive neuropsychological battery (RBANS) and to describe cut-off levels offering the optimal sensitivity and specificity in identifying different degrees of cognitive impairment due to the need to identify a greater number of patients with cognitive impairment [4]. Given the close relationship between cognitive impairment and impairment in other domains [22], the widespread use of MMSE, in particular, requires improvements in its use as a first-level screening tool.

The correlation between the two tests was moderate (r = 0.49), and MMSE, according to the most common 24-point cut-off score, only identified deficiency in less than 30% of the sample, while RBANS, on the contrary, showed only 25.2% of the sample to have adequate cognitive abilities. Moreover, MMSE’s internal consistency was questionable (Cronbach’s alpha: 0.63).

In order to discriminate mild from severe cognitive impairment, we considered the 85-point and the 70-point RBANS cut-off scores, respectively. We obtained a first AUC study to evaluate which MMSE score could offer the better sensitivity and specificity in identifying advanced cognitive deficit. The 24-point cut-off score demonstrated a good specificity and an acceptable sensitivity. The second AUC was studied to evaluate which MMSE score is better in identifying a milder cognitive deficit. The analysis of Youden’s J demonstrated that the better sensitivity/specificity balance was exhibited by the 29.7-point cut-off. Through the analysis, it emerged that even the consideration of lower scores leads to a poor increase in the specificity, with lowered sensitivity. This aspect emphasizes the poor capacity of MMSE for identifying people with low-degree cognitive impairment, because the 29.7-point cut-off has no clinical significance, owing to the fact that MMSE scores range from 0 to 30. For this reason, our final analysis was conducted considering the “impaired” subjects with both mild and severe impairment. The 26.1-point cut-off seemed to show an adequate performance in this sense. This result follows in the literature’s footsteps, which firstly focused on the 24-point cutoff [8,23,24], while more recent studies considered higher scores as the optimal cut-off, in particular the 26-point cut-off [25,26], though MMSE remains less sensitive than other tools, even though it is widely used [7].

The study of the thresholds of the four subsets according to age showed that people between 71 and 78 years old would benefit more from MMSE, while for people younger than 70, we found that 29.7 points is the best cut-off level (with no clinical significance, as stated before), and for people older than 78, we did not even find a significant cut-off level. These data confirm that MMSE is not the ideal tool for detecting an early deficit typical of younger people and that, paradoxically, it loses its meaning with increasing age, since aging is related to a “phenotypic manifestation” of NCD, making the screening tests less useful.

## 5. Conclusions

Our analysis showed that MMSE, though widely administered, is not able to identify early cognitive deficit, especially when considering the 24-point cut-off score. Different cut-offs are needed to discriminate between different impairment degrees, and the 26.1-point score seems to be preferable to the others, especially for people aged between 71 and 78.

The limitations of this study include the fact that it is monocentric and enrolled a relatively small sample. Further studies with longitudinal monitoring could confirm the suitability of our hypothesis and encourage the development of more sensitive first-level cognitive screening tests.

## Figures and Tables

**Table 1:** Characteristics of the sample.

Variables	Minimum	Maximum	Mean	SD
Age (years)	60	87	73.77	5.81
Years in school	0	18	8.04	4.49
RBANS total scale (corrected for age)	46	131	77.24	14.44
MMSE (corrected for age and years in school)	14.7	30	25.25	3.69
MMSE prientation	3	10	8.86	1.66
MMSE immediate memory	1	3	2.98	0.19
MMSE attention	0	5	4.33	1.25
MMSE delayed memory	0	3	1.78	1.13
MMSE language	4	9	7.94	1.11

MMSE, Mini-Mental State Examination; RBANS, Repeatable Battery for the Assessment of Neurocognitive Status; SD, standard deviation.

**Table 2:** Cronbach’s alpha—dropping variables.

Variable Dropped	Alpha	Change
MMSE Orientation	0.49	−0.14
MMSE Immediate Memory	0.67	0.04
MMSE Attention	0.54	−0.09
MMSE Delayed Memory	0.54	−0.09
MMSE Language	0.56	−0.07

MMSE, Mini-Mental State Examination.

**Table 3:** Criterion values and coordinates of the ROC curve (severe impairment).

Criterion	Sensitivity	95% C.I.	Specificity	95% C.I.	+LR	−LR
<14.7	0	0.0–4.6	100	98.0–100.0		1
≤15	3.8	0.8–10.7	100	98.0–100.0		0.96
≤15.3	3.8	0.8–10.7	99.45	97.0–100.0	6.95	0.97
≤18.9	24.05	15.1–35.0	99.45	97.0–100.0	44.01	0.76
≤19	25.32	16.2–36.4	98.91	96.1–99.9	23.16	0.76
≤19.3	25.32	16.2–36.4	98.36	95.3–99.7	15.44	0.76
≤19.5	27.85	18.3–39.1	98.36	95.3–99.7	16.99	0.73
≤19.7	32.91	22.7–44.4	97.81	94.5–99.4	15.06	0.69
≤20	35.44	25.0–47.0	97.81	94.5–99.4	16.22	0.66
≤20.1	35.44	25.0–47.0	97.27	93.7–99.1	12.97	0.66
≤21.1	41.77	30.8–53.4	97.27	93.7–99.1	15.29	0.6
≤21.4	41.77	30.8–53.4	96.72	93.0–98.8	12.74	0.6
≤21.5	43.04	31.9–54.7	96.72	93.0–98.8	13.13	0.59
≤21.9	43.04	31.9–54.7	96.17	92.3–98.4	11.25	0.59
≤22	44.3	33.1–55.9	93.99	89.5–97.0	7.37	0.59
≤22.2	44.3	33.1–55.9	93.44	88.8–96.6	6.76	0.6
≤22.4	48.1	36.7–59.6	92.35	87.5–95.8	6.29	0.56
≤22.7	53.16	41.6–64.5	91.26	86.2–94.9	6.08	0.51
≤22.9	54.43	42.8–65.7	91.26	86.2–94.9	6.23	0.5
≤23.2	54.43	42.8–65.7	90.16	84.9–94.1	5.53	0.51
≤23.3	55.7	44.1–66.9	88.52	83.0–92.8	4.85	0.5
≤23.4	55.7	44.1–66.9	87.98	82.4–92.3	4.63	0.5
≤23.6	58.23	46.6–69.2	87.98	82.4–92.3	4.84	0.47
≤23.7	60.76	49.1–71.6	85.25	79.3–90.0	4.12	0.46
≤24	63.29	51.7–73.9	84.7	78.7–89.6	4.14	0.43
≤24.2	63.29	51.7–73.9	83.06	76.8–88.2	3.74	0.44
≤24.3	64.56	53.0–75.0	81.42	75.0–86.8	3.47	0.44
≤24.4	65.82	54.3–76.1	80.33	73.8–85.8	3.35	0.43
≤24.5	67.09	55.6–77.3	80.33	73.8–85.8	3.41	0.41
≤24.7	68.35	56.9–78.4	77.05	70.3–82.9	2.98	0.41
≤24.9	68.35	56.9–78.4	75.96	69.1–82.0	2.84	0.42
≤25	69.62	58.2–79.5	73.77	66.8–80.0	2.65	0.41
≤25.3	69.62	58.2–79.5	70.49	63.3–77.0	2.36	0.43
≤25.4	70.89	59.6–80.6	70.49	63.3–77.0	2.4	0.41
≤25.7	74.68	63.6–83.8	67.76	60.5–74.5	2.32	0.37
≤25.8	74.68	63.6–83.8	67.21	59.9–74.0	2.28	0.38
≤25.9	75.95	65.0–84.9	65.03	57.6–71.9	2.17	0.37
≤26	77.22	66.4–85.9	61.75	54.3–68.8	2.02	0.37
≤26.1	77.22	66.4–85.9	60.66	53.2–67.8	1.96	0.38
≤26.2	78.48	67.8–86.9	58.47	51.0–65.7	1.89	0.37
≤26.3	78.48	67.8–86.9	53.55	46.0–60.9	1.69	0.4
≤26.4	81.01	70.6–89.0	50.27	42.8–57.7	1.63	0.38
≤26.7	83.54	73.5–90.9	46.45	39.1–54.0	1.56	0.35
≤26.8	84.81	75.0–91.9	46.45	39.1–54.0	1.58	0.33
≤26.9	84.81	75.0–91.9	45.9	38.5–53.4	1.57	0.33
≤27	86.08	76.5–92.8	41.53	34.3–49.0	1.47	0.34
≤27.3	86.08	76.5–92.8	36.07	29.1–43.5	1.35	0.39
≤27.4	91.14	82.6–96.4	32.79	26.0–40.1	1.36	0.27
≤27.7	94.94	87.5–98.6	30.05	23.5–37.3	1.36	0.17
≤28.4	94.94	87.5–98.6	22.4	16.6–29.1	1.22	0.23
≤28.7	96.2	89.3–99.2	20.77	15.1–27.4	1.21	0.18
≤29	98.73	93.1–100.0	20.22	14.7–26.8	1.24	0.063
≤29.7	98.73	93.1–100.0	19.13	13.7–25.6	1.22	0.066
≤30	100	95.4–100.0	0	0.0–2.0	1	

CI, confidence interval; +LR, positive likelihood ratio; −LR, negative likelihood ratio.

**Table 4:** Criterion values and coordinates of the ROC curve (mild impairment).

Criterion	Sensitivity	95% C.I.	Specificity	95% C.I.	+LR	−LR
<15.3	0	0.0–3.1	100	94.6–100.0		1
≤21.9	5.98	2.4–11.9	100	94.6–100.0		0.94
≤22	8.55	4.2–15.2	98.48	91.8–100.0	5.64	0.93
≤22.4	11.11	6.1–18.3	98.48	91.8–100.0	7.33	0.9
≤22.7	11.11	6.1–18.3	95.45	87.3–99.1	2.44	0.93
≤23	11.97	6.7–19.3	95.45	87.3–99.1	2.63	0.92
≤23.2	11.97	6.7–19.3	93.94	85.2–98.3	1.97	0.94
≤23.3	13.68	8.0–21.3	92.42	83.2–97.5	1.81	0.93
≤24.1	20.51	13.6–29.0	92.42	83.2–97.5	2.71	0.86
≤24.2	20.51	13.6–29.0	89.39	79.4–95.6	1.93	0.89
≤24.4	24.79	17.3–33.6	89.39	79.4–95.6	2.34	0.84
≤24.7	27.35	19.5–36.4	84.85	73.9–92.5	1.81	0.86
≤24.9	28.21	20.3–37.3	83.33	72.1–91.4	1.69	0.86
≤25	31.62	23.3–40.9	83.33	72.1–91.4	1.9	0.82
≤25.2	31.62	23.3–40.9	81.82	70.4–90.2	1.74	0.84
≤25.3	35.9	27.2–45.3	81.82	70.4–90.2	1.97	0.78
≤25.7	37.61	28.8–47.0	77.27	65.3–86.7	1.65	0.81
≤25.8	37.61	28.8–47.0	75.76	63.6–85.5	1.55	0.82
≤26.1	47.86	38.5–57.3	75.76	63.6–85.5	1.97	0.69
≤26.2	48.72	39.4–58.1	71.21	58.7–81.7	1.69	0.72
≤26.3	53.85	44.4–63.1	66.67	54.0–77.8	1.62	0.69
≤26.4	57.26	47.8–66.4	63.64	50.9–75.1	1.57	0.67
≤26.7	60.68	51.2–69.6	59.09	46.3–71.0	1.48	0.67
≤26.9	61.54	52.1–70.4	59.09	46.3–71.0	1.5	0.65
≤27	65.81	56.5–74.3	54.55	41.8–66.9	1.45	0.63
≤27.1	66.67	57.4–75.1	54.55	41.8–66.9	1.47	0.61
≤27.2	66.67	57.4–75.1	53.03	40.3–65.4	1.42	0.63
≤27.3	72.65	63.6–80.5	51.52	38.9–64.0	1.5	0.53
≤27.4	76.07	67.3–83.5	48.48	36.0–61.1	1.48	0.49
≤27.7	79.49	71.0–86.4	46.97	34.6–59.7	1.5	0.44
≤28	81.2	72.9–87.8	43.94	31.7–56.7	1.45	0.43
≤28.1	82.05	73.9–88.5	42.42	30.3–55.2	1.43	0.42
≤28.3	83.76	75.8–89.9	42.42	30.3–55.2	1.45	0.38
≤28.4	85.47	77.8–91.3	36.36	24.9–49.1	1.34	0.4
≤29.7	90.6	83.8–95.2	36.36	24.9–49.1	1.42	0.26
≤30	100	96.9–100.0	0	0.0–5.4	1	

CI, confidence interval; +LR, positive likelihood ratio; −LR, negative likelihood ratio.

**Table 5:** Criterion values and coordinates of the ROC curve (mild and severe impairment).

Criterion	Sensitivity	95% C.I.	Specificity	95% C.I.	+LR	−LR
<14.7	0	0.0–1.9	100	94.6–100.0		1
≤21.9	20.92	15.4–27.3	100	94.6–100.0		0.79
≤22	22.96	17.3–29.5	98.48	91.8–100.0	15.15	0.78
≤22.4	26.02	20.0–32.8	98.48	91.8–100.0	17.17	0.75
≤22.7	28.06	21.9–34.9	95.45	87.3–99.1	6.17	0.75
≤23	29.08	22.8–36.0	95.45	87.3–99.1	6.4	0.74
≤23.2	29.08	22.8–36.0	93.94	85.2–98.3	4.8	0.75
≤23.3	30.61	24.2–37.6	92.42	83.2–97.5	4.04	0.75
≤24.1	37.76	30.9–44.9	92.42	83.2–97.5	4.98	0.67
≤24.2	37.76	30.9–44.9	89.39	79.4–95.6	3.56	0.7
≤24.5	41.84	34.8–49.1	89.39	79.4–95.6	3.94	0.65
≤24.7	43.88	36.8–51.1	84.85	73.9–92.5	2.9	0.66
≤24.9	44.39	37.3–51.6	83.33	72.1–91.4	2.66	0.67
≤25	46.94	39.8–54.2	83.33	72.1–91.4	2.82	0.64
≤25.2	46.94	39.8–54.2	81.82	70.4–90.2	2.58	0.65
≤25.4	50	42.8–57.2	81.82	70.4–90.2	2.75	0.61
≤25.7	52.55	45.3–59.7	77.27	65.3–86.7	2.31	0.61
≤25.8	52.55	45.3–59.7	75.76	63.6–85.5	2.17	0.63
≤26.1	59.69	52.5–66.6	75.76	63.6–85.5	2.46	0.53
≤26.2	60.71	53.5–67.6	71.21	58.7–81.7	2.11	0.55
≤26.3	63.78	56.6–70.5	66.67	54.0–77.8	1.91	0.54
≤26.4	66.84	59.8–73.4	63.64	50.9–75.1	1.84	0.52
≤26.7	69.9	63.0–76.2	59.09	46.3–71.0	1.71	0.51
≤26.9	70.92	64.0–77.2	59.09	46.3–71.0	1.73	0.49
≤27	73.98	67.2–80.0	54.55	41.8–66.9	1.63	0.48
≤27.1	74.49	67.8–80.4	54.55	41.8–66.9	1.64	0.47
≤27.2	74.49	67.8–80.4	53.03	40.3–65.4	1.59	0.48
≤27.3	78.06	71.6–83.6	51.52	38.9–64.0	1.61	0.43
≤27.4	82.14	76.1–87.2	48.48	36.0–61.1	1.59	0.37
≤27.7	85.71	80.0–90.3	46.97	34.6–59.7	1.62	0.3
≤28	86.73	81.2–91.1	43.94	31.7–56.7	1.55	0.3
≤28.1	87.24	81.7–91.6	42.42	30.3–55.2	1.52	0.3
≤28.3	88.27	82.9–92.4	42.42	30.3–55.2	1.53	0.28
≤28.4	89.29	84.1–93.2	36.36	24.9–49.1	1.4	0.29
≤29.7	93.88	89.5–96.8	36.36	24.9–49.1	1.48	0.17
≤30	100	98.1–100.0	0	0.0–5.4	1	

CI, confidence interval; +LR, positive likelihood ratio; −LR, negative likelihood ratio.

**Table 6:** Youden’s indexes of the subsets.

Quartile	Criterion	J	Sensitivity	95% C.I.	Specificity	95% C.I.	AUC	*p*
1	≤29.7	0.36	88.89	75.9–96.3	46.88	29.1–65.3	0.71	**0.0005**
2	≤23.7	0.36	41.46	26.3–57.9	94.44	72.7–99.9	0.69	**0.0058**
3	≤26	0.67	67.24	53.7–79.0	100.00	66.4–100.0	0.84	**<0.0001**
4	≤28	0.49	92.31	81.5–95.7	57.14	18.4–90.1	0.72	0.0809

CI, confidence interval; AUC, area under the ROC curve.

## Data Availability

The data and materials used and/or analyzed during the current study are not publicly available. They are available from the corresponding author upon reasonable request.

## Abbreviations

AUC: area under the ROC curve; CI: confidence interval; +LR: positive likelihood ratio; −LR: negative likelihood ratio; MMSE: Mini-Mental State Examination; MoCA: Montreal Cognitive Assessment; RBANS: Repeatable Battery for the Assessment of Neuropsychological Status; RBANS-IS: RBANS index score; RBANS-TIS: RBANS total scale index; ROC: receiver operating characteristic; SD: standard deviation.
